# Comparison of Dosimetric Gains Provided by Intensity-Modulated Radiotherapy, Volume-Modulated Arc Therapy, and Helical Tomotherapy for High-Grade Glioma

**DOI:** 10.1155/2020/4258989

**Published:** 2020-03-18

**Authors:** Pei Liu, Gui Liu, Guihua Wang, Weibing Zhou, Yangqing Sun, Wen Chen, Qian Zeng, Jidong Hong, Qiongxuan Xie, Ludi Ou, Rui Wei

**Affiliations:** ^1^Department of Oncology, Xiangya Hospital, Central South University, Changsha, Hunan, China 410008; ^2^National Clinical Research Center for Geriatric Disorders, Xiangya Hospital, Central South University, Changsha, Hunan, China 410008; ^3^Department of Oncology, Changsha Central Hospital, Changsha, Hunan, China 410008

## Abstract

**Purpose:**

Because of the poor prognosis for high-grade glioma (HGG) patients, it is important to increase the dose of the tumor to improve the efficacy while minimizing the dose of organs at risk (OARs). Thus, we evaluated the potential dosimetric gains of helical tomotherapy (HT) versus intensity-modulated radiotherapy (IMRT) or volume-modulated arc therapy (VMAT) for high-grade glioma (HGG).

**Methods:**

A total of 42 HGG patients were retrospectively selected who had undergone helical tomotherapy; then, IMRT and VMAT plans were generated and optimized for comparison after contouring crucial neuronal structures for neurogenesis and neurocognitive function. IMRT and VMAT were optimized with the Eclipse treatment planning system (TPS) (Version 11.0.31) and HT using TomoTherapy Hi-Art Software (Version 2.0.7) (Accuray, Madison, WI, USA). All three techniques were optimized for simultaneously delivering 60 Gy to planning target volume (PTV) 1 and 50-54 Gy to PTV2. We also analyzed the homogeneity index (HI) and conformity index (CI) of PTVs and organ at risk (OAR) sparing.

**Results:**

There was no significant difference in the PTV coverage among IMRT, VMAT, or HT. As for the HI, HT plans (PTV1 HI: 0.09 ± 0.03, PTV2 HI: 0.17 ± 0.05) had the best homogeneity when compared to IMRT plans (PTV1 HI: 0.10 ± 0.04, PTV2 HI: 0.18 ± 0.04) and VMAT plans (PTV1 HI: 0.11 ± 0.03, PTV2 HI: 0.20 ± 0.03). The CI value of HT (PTV1 CI: 0.98 ± 0.03, PTV2: 0.98 ± 0.05) was closest to the optimal value. Except for the IMRT and VMAT groups, there were statistically significant differences between the other two groups of the CI values in both PTV1 and PTV2. The other comparison values were statistically significant except for the optic nerve, and VMAT had the best sparing of the optic chiasm. The mean and max doses of OARs declined significantly in HT.

**Conclusions:**

For high-grade glioma patients, HT had superior outcomes in terms of PTV coverage and OAR sparing as compared with IMRT/VMAT.

## 1. Introduction

Gliomas are tumors that originate from glial cells and have high morbidity, high recurrence, and poor prognosis. One study showed that gliomas represent 47.1% of primary malignant brain and other central nervous system tumors, of which glioblastoma is the main type of gliomas, accounting for approximately 56.1% of cases [1]. The treatment process includes surgery, followed by radiotherapy with or without temozolomide chemotherapy [2]. Due to the biological characteristics of the tumor at the site and the limitations of the anatomical site, most tumors require radiation therapy after surgery. Radiotherapy (RT) occupies an integral role in treating gliomas [3, 4], and survival is significantly reduced in glioblastoma patients if RT is not initiated within the 6 weeks after complete resection of the tumor [5].

Advances in radio physical technology have led to better radiotherapy techniques that increase the dose of the target volume while reducing the dose of the surrounding normal tissue. These radiotherapeutic techniques include intensity-modulated radiation therapy (IMRT), volumetric-modulated radiation arc therapy (VMAT), and Hi-Art helical tomotherapy (HT) [6, 7]. These mentioned techniques have helped to balance planning target volume (PTV) coverage and organ at risk (OAR) sparing.

After two-dimensional radiotherapy (2DCRT) and three-dimensional conformal radiotherapy (3DCRT), IMRT has become an important radiotherapy technique for head and neck tumors like nasopharyngeal carcinoma [8], because it has been demonstrated to improve tumor coverage while reducing the dose of the surrounding tissues [9–11]. IMRT yielded excellent survival outcomes compared to 2DCRT or 3DCRT [12]. Meanwhile, the security boundary setting should be different according to different anatomic sites changing during the course of radiotherapy [13].

IMRT is a type of high-precision radiotherapy that uses computer-controlled linear accelerators to provide precise radiation doses for malignancies or advanced patterns in specific areas. However, IMRT has shortcomings that cannot be ignored. For one, due to the high number of monitor units (MUs) and long delivery time, the IMRT efficiency is relatively low, thus reducing the target biological dose [14, 15]. For another, increased beams and a high number of MUs allow more normal tissues to receive radiation, thus elevating the risks of secondary tumors in patients [16, 17].

In order to improve the inherent defects of IMRT, VMAT was developed. Its biggest advantage is to shorten the MUs and improve treatment efficiency [18]. The features of a 360° multiarc setting for rotating illumination in any angular range and continuous dynamic changes in parameters such as rack speed, dose rate, and multileaf collimator (MLC) position may contribute to this progress [19, 20].

With the advancement of technology, HT was first used in clinical practice in 2003. The basic principle of HT is to install a 6 MV linear accelerator on a special computed tomography (CT) slip ring frame for 360° rotation, using a fan-shaped X-ray beam for tomographic illumination. It integrates intensity-modulated radiotherapy (IMRT), image-guided intensity-modulated radiotherapy (IGRT), and dose-guided intensity-modulated radiotherapy (DGRT). Through the megavolt images obtained from each treatment, tumor dose distribution and tumor during treatment can be observed. The treatment plan can be changed, and the target volume can be adjusted in a timely fashion [21]. The ability to treat different target areas, from the stereotactic treatment of small tumors to systemic treatment, is performed by a single spiral beam.

This study is aimed at evaluating the potential dosimetric gains of helical tomotherapy (HT) versus intensity-modulated radiotherapy (IMRT) or volume-modulated arc therapy (VMAT) for high-grade glioma (HGG).

## 2. Materials and Methods

### 2.1. Patient Characteristics

We conducted a retrospective study on 42 consecutive patients who had undergone postoperative HT for histologically proven HGG, including anaplastic astrocytoma, anaplastic oligodendroglioma, or glioblastoma at the Department of Radiation Oncology, Xiangya Hospital Central South University between October 2015 and March 2018. All patients underwent a maximum reasonable microsurgical resection. None of them had distant metastases or received any previous RT. Patients were informed of the course of radiotherapy and potential risks and signed informed consent. Patient characteristics are listed in Table 1.

### 2.2. Treatment Planning

The patients assumed a supine position with arms on either side of the body and with a thermoplastic head-shoulder mask on the scanning bed. All subjects were scanned by a Siemens Plus 4 Spiral computed tomography (CT) simulator for 3 mm slice thicknesses. Scanning started from head overhead to 5 cm below the occipital foramen, with MRI scanning in the same fixed position. Then, CT and magnetic resonance imaging (MRI) images were imported into the Eclipse TPS (Varian Medical Systems Inc., Version 11.0.31); next, fusion CT and MRI images were captured so that clinicians could delineate the target area.

### 2.3. Statistical Analyses

Analysis of variance (ANOVA) was performed on all data using SPSS statistical software (Version 20.0), with a comparison between two pairs using the least significant difference (LSD) method. The dosimetric differences in two of the three groups were compared, with *p* < 0.05 being considered statistically significant.

### 2.4. Target Volume Delineation and Dose Prescription

The target and the critical organ volumes in all cases were outlined by the same oncologist (with workstation). The target volumes were defined following the European Organization for Research and Treatment of Cancer (EORTC).

The gross tumor volume (GTV) included the MRI T1 enhancement zone and surgery cavity, while it excluded the peritumoral edema zone. GTV expanded the two-centimeter isometric margin to obtain clinical target volume 1 (CTV1) and expanded centimeter isometric margin to obtain clinical target volume 2 (CTV2). A planned target volume (PTV) with a margin of 3 mm is typically added to the CTV. OARs included the brain stem, lens, optic nerves, optic chiasm, pituitary, and other nontarget tissues. All the three plans were given to the same prescription dose and division method. The dose prescriptions were 60 Gy/30 f to PTV1 and 50-54 Gy/30 f to PTV2. 100% of the prescribed dose would cover 95% of planning target volume (PTV) with a maximum dose (*D*_max_) of <110%. The restriction of the organ at risk (OAR) dose was determined according to the ESTRO-ACROP [22] as follows: brain stem, *D*_max_ ≤ 54 Gy; both optic nerves and optic chiasm, *D*_max_ < 55 Gy; pituitary, *D*_max_ < 50Gy; and lens, the smaller the *D*_max_, the better. Parameters and priorities were constantly adjusted under the premise of preferentially meeting target coverage, minimizing the risk, and optimizing the results, in order to implement a more reasonable treatment plan (Table 2).

A 6 MV linear accelerator made all plans. HT plans were drawn up on TomoTherapy Hi-Art Software (Version 2.0.7) (Accuray, Madison, WI, USA), while IMRT and VMAT plans were drafted on Varian Eclipse TPS (Varian Medical System, USA). These three plans were all redesigned by the same physicist.

### 2.5. Radiation Techniques

We followed the methods of Chen et al. [23].

#### 2.5.1. IMRT

IMRT plans were made of 6 coplanar fields (6F-IMRT). A medical physicist would commission the position, size, and angle of the collimator with the same thermoplastic head mask. The Dose Volume Optimizer (Varian Eclipse, Version 11.0.31) algorithm of Eclipse TPS was used for plan optimization. The plans were iteratively optimized by inverse planning software for optimal PTV coverage and OAR sparing. There were 60 leaves on one side of the accelerator, of which 40 leaves were located in the middle, each width was 0.5 cm, and 10 leaves were located on both sides, each width was 1.0 cm. Final dose distribution was calculated by Anisotropic Analytical Algorithm (AAA, Version 11.031) dosage algorithm with a calculation grid size of 2.5 mm.

#### 2.5.2. VMAT

VMAT plans were produced by using two complementary coplanar arcs of 360° (one counterclockwise from 179° to 181° and the other clockwise from 181° to 179°). The medical physicist would optimize the VMAT plan by progressive resolution of Eclipse TPS (Version 11.0.31). The VMAT plans used the same accelerator as IMRT and had the same optimization objectives as the 6F-IMRT plans. Other planning parameters contained MLC motion speed 0 to 2.5 cm/s, a gantry rotation speed of 0.5 to 4.8 degrees/s, and a dosing rate of 0 to 600 MU/min. The final dose distribution was calculated by the AAA algorithm with a grid size of 2.5 mm.

#### 2.5.3. HT

HT plans were optimized using TomoTherapy Hi-Art software (Version 2.0.7) (Accuray, Madison, WI, USA). The operator sets the three main parameters as the following: field width, 2.5 cm; pitch, 0.287; and modulation factor, 2.1–2.6. Dose calculations were performed using a collapsed cone convolution model with a grid size of 1.95 mm.

### 2.6. Plan Evaluations

The three plans evaluated the quality and quantity by using dose-volume histograms (DVHs). According to the criteria of the International Commission on Radiation Units and Measurements 83 report (ICRU83), the near-maximum (D2%), near-minimum (D98%), and median (D50%) doses were used to assess the conformity index (CI) and homogeneity index (HI). The criteria of the dosimetric comparison were as follows: HI = (D2% − D98%)/D50%, where HI indicates the homogeneity of the plan and the optimal value is zero; that is, a higher HI means worse homogeneity.

CI = (VRT/VT) × (VRT/VR), where VRT is the volume of PTV covered by 95% of the prescribed dose, VT is the volume of the target, and VR is the total volume of the body covered by the prescribed dose; CI had a range from 0 to 1 with an optimal value of 1.

OAR maximal dose (*D*_max_) and mean dose (*D*_mean_) were determined for the brain stem PRV, lenses, optic nerves, optic chiasm, and the pituitary; the lower the value is, the better the protection.

## 3. Results

### 3.1. PTV Coverage (V95) and Conformality

**Figure 1:**
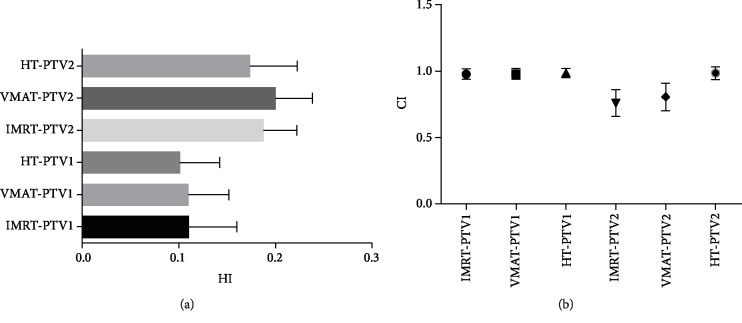
(a) The value of HI for PTV1 and PTV2. (b) The value of CI for PTV1 and PTV2. PTV: planning target volume; IMRT: intensity-modulated radiation therapy; VMAT: volumetric-modulated arc therapy; HT: helical tomotherapy.

**Figure 2:**
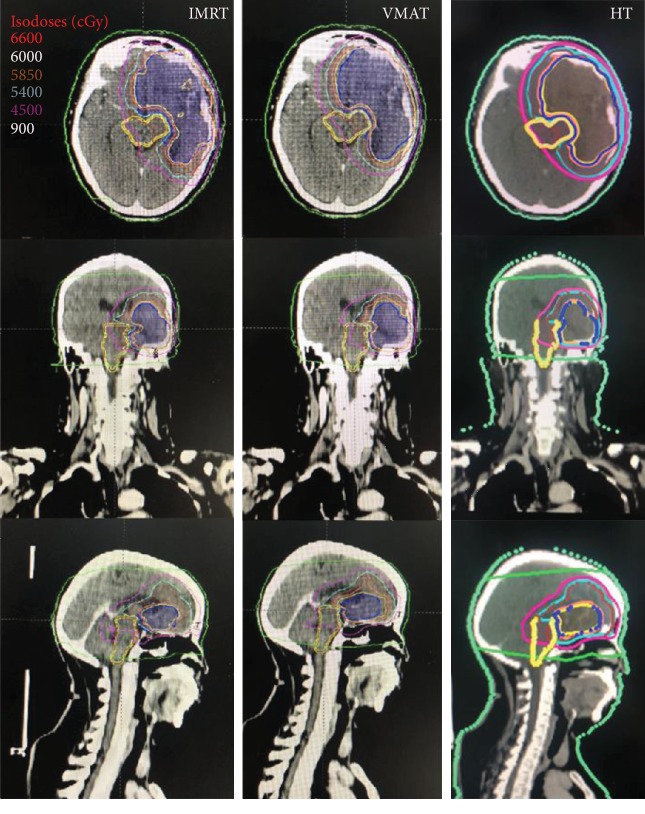
One patient's dose distributions on IMRT, VMAT, and HT. Color-wash areas: 66.00 Gy (red), 60.00 Gy (yellow), 58.50 Gy (orange), 54.00 Gy (cyan), 45.00 Gy (purple), and 9 Gy (green). IMRT: intensity-modulated radiation therapy; VMAT: volumetric-modulated arc therapy; HT: helical tomotherapy.

The dosimetric data and conformality parameters are tabulated in Table 3 and Figure 1. The coverage of all three planned PTVs was assessed by comparing the target volumes receiving 95% of the prescribed dose (V95%). In PTV1, the V95% for IMRT, VMAT, and HT was 98.37% ± 3.45%, 98.46% ± 3.28%, and 98.52% ± 3.66%, respectively. In PTV2, the V95% values were 97.56 ± 3.26%, 97.81 ± 2.96%, and 98.26 ± 5.27% in IMRT, VMAT, and HT, respectively. All plans had similar PTV coverage and were subject to prescription requirements. There was no significant difference in the target coverage among IMRT, VMAT, and HT plans.  Figure 2 shows the typical dose distributions produced by each of the three techniques.

As for HI, HT plans (PTV1 HI: 0.09 ± 0.03, PTV2 HI: 0.17 ± 0.05) had the best homogeneity when compared to IMRT plans (PTV1 HI: 0.10 ± 0.04, PTV2 HI: 0.18 ± 0.04) and VMAT plans (PTV1 HI: 0.11 ± 0.03, PTV2 HI: 0.20 ± 0.03). However, there was no statistically significant difference between HT and IMRT or VMAT and IMRT.

The CI value of HT (PTV1 CI: 0.98 ± 0.03, PTV2: 0.98 ± 0.05) was closest to the optimal value, while the CI of IMRT was PTV1 CI: 0.97 ± 0.04 and PTV2 CI: 0.76 ± 0.10, and that of VMAT was PTV1 CI: 0.97 ± 0.04 and PTV2 CI: 0.80 ± 0.10. Except for the statistical difference between the CI values of IMRT and VMAT in PTV1, the other comparison values were statistically significant.

### 3.2. Dose to OARs

**Figure 3:**
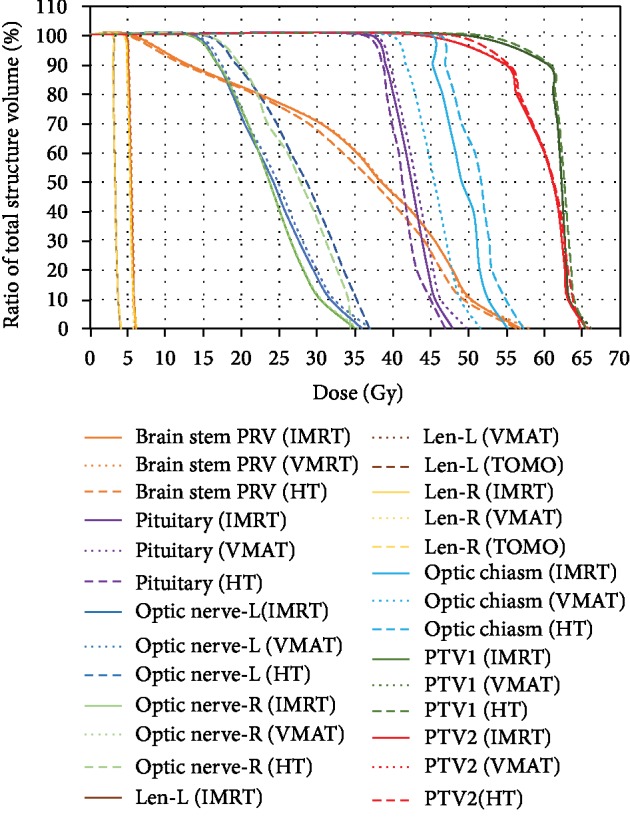
The average DVH to the OARs of 42 HGG patients. DVH: dose-volume histogram; OARs: organs at risk; PTV: planning target volume; PRV: planning risk volume; Len-L: left lens; Len-R: right lens; Optic nerve-L: left optic nerve; Optic nerve-R: right optic nerve; IMRT: intensity-modulated radiation therapy; VMAT: volumetric-modulated arc therapy; HT: helical tomotherapy.

**Figure 4:**
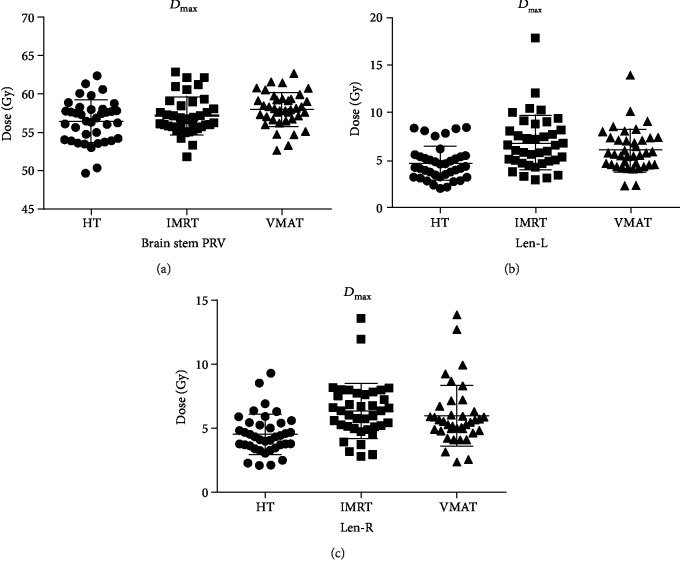
(a) The *D*_max_ dose of brain steam PRV. (b) The *D*_max_ dose of left lens. (c) The *D*_max_ dose of right lens. *D*_max_: maximum point dose of the volume; Len-L: left lens; Len-R: right lens; IMRT: intensity-modulated radiation therapy; VMAT: volumetric-modulated arc therapy; HT: helical tomotherapy; *D*_max_: maximum point dose of the volume.

Average DVHs for PTV and selected OARs are presented in Figure 3, and the dosimetric comparison for OARs is shown in Table 4 and Figure 4.

### 3.3. PRV of Brain Stem (Brain Stem with 1-Millimeter Extension)

HT allowed more sparing of the PRV brain stem than IMRT and VMAT. *D*_mean_ was significantly lower in the case of HT (*p* = 0.018 and *p* = 0.029, respectively). As for *D*_max_, both IMRT and VMAT doses were higher than those of HT (VMAT vs. HT (*p* = 0.009); IMRT vs. HT (*p* = 0.046)).

### 3.4. Lens

Whether it was *D*_max_ or *D*_mean_, there was a statistically significant difference between HT and the other two groups (*p* ≤ 0.001, *p* = 0.011), which could reduce the dose by approximately 1 to 2 Gy. However, for IMRT and VMAT, no statistically significant difference was observed for both the left and right lenses.

### 3.5. Optic Nerves

The dose of *D*_max_ was the lowest in HT while that of *D*_mean_ was the highest, while there was no statistical significance among these groups. All dose plans were within the planned objectives.

### 3.6. Optic Chiasm

VMAT achieved the largest dose reduction which could reduce the *D*_max_ of the optic chiasm by 4 to 6 Gy in general.

### 3.7. Pituitary

The maximal and mean doses to the pituitary in HT were slightly lower than those in IMRT or VMAT. No significant difference existed in IMRT/HT, while a statistical significance was found in VMAT/HT.

## 4. Discussion

The survival for glioblastoma is low with median survival being around one year and the five-year survival rate being <10% after diagnosis [24]; high-grade gliomas (HGGs) have an obvious tumor occupying effect and rich blood supply, with cells often showing invasive growth. It is therefore difficult to remove through surgery. Consequently, surgery combined with radiotherapy has become an important treatment for glioma. Studies have shown that postoperative radiotherapy for glioma can significantly prolong the survival of patients [25].

IMRT and VMAT have been widely used in clinical practice. HT is receiving attention as one of the new radiotherapy technologies. However, there are still few studies comparing HT, IMRT, and VMAT for glioma. In this study, the authors intended to evaluate the potential dosimetric gains of HT versus IMRT or VMAT, with the hope of providing a basis for the choice of treatment in the clinic.

We selected patients with gliomas who had undergone helical tomotherapy, and then IMRT and VMAT plans were generated and optimized. All the plans were subject to prescription requirements. In our study, the conformity of IMRT and VMAT was the same; except for the statistical difference between the CI values of PTV2 (*p* = 0.029), the others are not statistically significant. HT plans significantly improved target dose conformity and homogeneity, and HT had a steeper DVH (Figures 1 and 3). Zhang et al. [26] examined the dosimetric differences between HT and conventional medical linear accelerator-based IMRT (LIMRT) and concluded that HT plans provide better PTV coverage, homogeneity, and better normal tissue sparing.

As shown in Table 3, HT had the best value of V95%, but no significant difference was found in the target coverage between IMRT, VMAT, and HT plans (V95% of PTV1: IMRT vs. HT (*p* = 0.672), IMRT vs. VMAT (*p* = 0.516), and HT vs. VMAT (*p* = 0.913)) (V95% of PTV2: IMRT vs. HT (*p* = 0.397, IMRT vs. VMAT (*p* = 0.317), and HT vs. VMAT (*p* = 0.528)). All three plans had sufficient tumor coverage. HT plans improved the CI of PTVs, with PTV2 having a marked effect. Analogous results have already been published in several series of studies. Sun et al. [27] reported the dosimetric comparisons of 12 newly histologically diagnosed intracranial medulloblastoma patients using HT, VMAT, and IMRT and found that HT showed superior dose conformity as compared with IMRT and VMAT in PTVs. As discussed by Zhang et al. [28], the CI for PTV 59.4 was similar among the 4 techniques (TomoDirect, HT, VMAT, and fixed-field intensity-modulated radiotherapy), and the CI for PTV 50.4 showed a statistical significance of HT.

As for HI, HT plans still had the best homogeneity when compared to the other two plans for PTVs. It had no statistical significance between HT and IMRT (PTV1: IMRT vs. HT (*p* = 0.274), VMAT vs. HT (*p* = 0.035); PTV2: IMRT vs. HT (*p* = 0.231), VMAT vs. HT (*p* = 0.012)). This is slightly different from some other studies. Koca et al. [29] analyzed the potential dosimetric gains of HT versus the linear accelerator for 21 GBM patients; D98% and mean doses for simultaneous integrated boost (SIB) volume (PTV60) of HI showed statistically significant superiority to the linear accelerator (LINAC) (*p* < 0.0001). The reasons for the difference may be the following: (i) the sample size of the study was larger than that of Koca et al., and the statistical difference in some values may not be obvious when the sample size is increased; (ii) IMRT plans were made by a different number of coplanar fields. Rong et al. [30] compared the doses of HT, IMRT, and RapidArc in three radiotherapy techniques in whole brain radiotherapy and also obtained better uniformity of HT.

In this study, with regard to the dose to OARs, VMAT and HT provided better sparing of OARs than IMRT. HT could maximally reduce the dose to the brain stem PRV, lens, and *D*_max_ of the optic nerves and pituitary. This advantage is most apparent in the brain stem PRV and lens. It can be clearly seen from Table 4 and Figure 4 that the dose of HT is lower than those of the other two groups. Brain stem PRV and the dose of HT (*D*_max_ = 55.62 ± 5.52; *D*_mean_ = 34.14 ± 6.52) were about 1 to 2 Gy lower than those in IMRT (*D*_max_ = 56.74 ± 3.57; *D*_mean_ = 36.67 ± 6.04) or VMAT (*D*_max_ = 57.57 ± 3.43; *D*_mean_ = 36.02 ± 6.15), and the difference was statistically significant. Chen et al. [23] analyzed the potential dosimetric gains of IMRT, VMAT, and HT for 30 locally advanced NPC patients. They found that HT could significantly reduce the dose to the brain stem (IMRT vs. HT: *p*_*D*max_ < 0.001, *p*_*D*mean_ < 0.001; VMAT vs. HT: *p*_*D*max_ < 0.05 or *p*_*D*mean_ < 0.001) and lens (IMRT vs. HT: *p*_*D*max_ < 0.001, *p*_*D*mean_ < 0.001; VMAT vs. HT: *p*_*D*max_ < 0.001 or *p*_*D*mean_ < 0.001) as compared with VMAT/IMRT. Our study is consistent with the above report.

For the *D*_mean_ of the optic nerves and optic chiasm, HT resulted in a higher dose than that of VMAT and IMRT. Meanwhile, VMAT had the minimum dose to the optic chiasm, but this is meaningless because the *D*_max_ doses of these three groups were far lower than the limit of 54 Gy. Li et al. [31] analyzed dosimetric differences between VMAT and HT for early T-stage nasopharyngeal carcinoma and found that VMAT showed significant superiority in sparing the optic nerves and optic chiasm. This is in line with our study. Another study [32] explored the dosimetric comparison of stage I-II nasal natural killer T cell lymphoma; VMAT and IMRT reduced the maximum dose of the optic chiasm (*p* = 0.016 and *p* = 0.020, respectively) when compared with HT. As for the pituitary, IMRT and HT were better than VMAT. Cao et al. [33] compared 10 treatment plans for patients who had originally undergone helical tomotherapy (3 head-and-neck cases, 2 cases each of prostate and lung cancer, and 1 case each of the brain, esophagus, and rectal cancer), using intensity-modulated arc therapy (IMAT) and HT. They concluded that HT can provide improved dosimetric results in the most complex cases.

From this statistical analysis of the data, HT can more effectively protect endangered organs while meeting target doses. Furthermore, a more tangible dose distribution can be obtained. Similar studies on tumors in other systems have shown that HT is superior to IMRT or VMAT or 3D-conformal radiotherapy in OARs, conformity, and homogeneity [34, 35].

Radiotherapy plans should be developed in the clinic to increase the irradiation dose in the treatment area and reduce the range of the normal tissue in the irradiated area. It is better to have lower acute or chronic toxicity, a higher tumor cure rate, and improved patient survival. This study only compared the dose of the above three radiotherapy techniques, aimed at providing some basis for the choice of radiotherapy in clinical practice. The next step is to further supplement the acute or chronic toxicity of radiotherapy and the subsequent survival rate of patients.

In conclusion, for high-grade glioma patients in the situation described above, HT has superior outcomes in terms of homogeneity, conformity, and OAR sparing as compared with IMRT/VMAT. Whether HT can prolong the survival, however, remains to be further studied. With the existing treatment technologies, the survival rate of glioma has not greatly improved. Our follow-up treatment methods can be applied to the update of radiotherapy technology and perhaps develop a more effective targeted treatment.

## Figures and Tables

**Table 1:** Patient characteristics.

Characteristics	*N*	(%)
Sex		
Male	27	64.28
Female	15	35.72
Median age in years (range)	46.56	
Initial diagnosis		
WHO III	24	57.14
WHO IV	18	42.86
Tumor localization		
Temporal	15	35.71
Frontal	14	33.33
Occipital	7	16.66
Parietal	6	14.30
Side of tumor localization		
Right	18	42.85
Left	23	54.76
Bilateral (central)	1	2.39

**Table 2:** Dose constraints for the critical structures and target volumes.

Structure	Dose constraints
Targets	
Maximum dose	<110% prescribed dose
Coverage V100%	≥95% PTV
OARs	
Brain stem	*D*_max_ ≤ 54 Gy
Lens	Ideally <6 Gy, max 10 Gy
Optic nerves	*D*_max_ ≤ 54 Gy
Optic chiasm	*D*_max_ < 55 Gy
Pituitary	*D*_max_ < 50 Gy

OARs: organs at risk.

**Table 3:** Dosimetric comparison for PTV1 and PTV2.

Target	IMRT	VMAT	HT	*p* value		
				IMRT vs. VMAT	IMRT vs. HT	VMAT vs. HT
PTV1						
D2% (Gy)	64.11 ± 0.92	64.63 ± 0.78	64.54 ± 0.65	0.011	0.079	0.419
D50% (Gy)	62.33 ± 0.66	62.75 ± 0.55	62.63 ± 0.80	0.020	0.252	0.230
D98% (Gy)	57.69 ± 2.04	57.85 ± 1.64	58.53 ± 2.13	0.632	0.119	0.277
*D*_max_ (Gy)	65.71 ± 1.13	66.28 ± 1.06	65.58 ± 1.56	0.038	0.897	0.028
*D*_min_ (Gy)	48.51 ± 4.38	50.52 ± 5.46	53.17 ± 5.21	0.048	≤0.001	0.040
V95 (%)	98.37 ± 3.45	98.46 ± 3.28	98.52 ± 3.66	0.516	0.672	0.913
CI	0.97 ± 0.04	0.97 ± 0.04	0.98 ± 0.03	0.728	0.042	0.009
HI	0.10 ± 0.04	0.11 ± 0.03	0.09 ± 0.03	0.483	0.274	0.035
PTV2						
D2% (Gy)	63.88 ± 0.83	64.45 ± 0.79	64.20 ± 0.92	0.097	0.393	0.013
D50% (Gy)	61.21 ± 1.40	61.42 ± 1.31	60.93 ± 1.50	0.519	0.071	0.015
D98% (Gy)	52.44 ± 1.73	52.50 ± 1.84	53.10 ± 1.93	0.828	0.633	0.795
*D*_max_ (Gy)	65.73 ± 1.14	66.03 ± 2.19	64.84 ± 2.48	0.598	0.138	0.045
*D*_min_ (Gy)	42.48 ± 5.57	42.48 ± 8.58	47.79 ± 9.48	0.936	0.006	0.008
V95 (%)	97.56 ± 3.26	97.81 ± 2.96	98.26 ± 5.27	0.317	0.397	0.528
CI	0.76 ± 0.10	0.80 ± 0.10	0.98 ± 0.05	0.029	≤0.001	≤0.001
HI	0.18 ± 0.04	0.20 ± 0.03	0.17 ± 0.05	0.175	0.231	0.012

PTV: planning target volume; D_*x*%_: dose received by *x*% of structure volume; *D*_max_: maximum point dose of the volume; *D*_min_: minimum point dose of the volume; V95%: volume receiving 95% of prescribed dose; IMRT: intensity-modulated radiation therapy; VMAT: volumetric-modulated arc therapy; HT: helical tomotherapy.

**Table 4:** Analysis of OAR doses.

OARs	Parameter	IMRT	VMAT	HT	*p* value		
					IMRT vs. VMAT	IMRT vs. HT	VMAT vs. HT
Brain stem PRV	*D*_max_	56.74 ± 3.57	57.57 ± 3.43	55.62 ± 5.52	0.143	0.046	0.009
	*D*_mean_	36.67 ± 6.04	36.02 ± 6.15	34.14 ± 6.52	0.629	0.018	0.029

Len-L	*D*_max_	6.48 ± 2.35	5.96 ± 2.18	4.56 ± 1.73	0.179	≤0.001	0.011
	*D*_mean_	5.39 ± 1.97	4.78 ± 1.54	3.48 ± 0.96	0.083	≤0.001	≤0.001

Len-R	*D*_max_	6.21 ± 2.29	5.87 ± 2.50	4.44 ± 1.67	0.476	≤0.001	0.004
	*D*_mean_	5.03 ± 1.92	4.62 ± 1.59	3.41 ± 1.14	0.305	≤0.001	0.001

Optic nerve-L	*D*_max_	37.91 ± 19.04	38.84 ± 19.27	38.46 ± 18.48	0.950	0.981	0.931
	*D*_mean_	25.93 ± 14.27	25.73 ± 13.79	27.46 ± 14.82	0.845	0.401	0.518

Optic nerve-R	*D*_max_	34.56 ± 18.57	34.17 ± 19.29	32.67 ± 18.65	0.992	0.736	0.743
	*D*_mean_	22.64 ± 13.84	23.24 ± 14.29	25.90 ± 14.55	0.876	0.316	0.379

Optic chiasm	*D*_max_	45.62 ± 11.11	41.03 ± 11.07	47.06 ± 7.63	0.027	0.594	0.009
	*D*_mean_	32.13 ± 15.37	28.84 ± 14.43	35.2 ± 10.92	0.017	0.062	0.039

Pituitary	*D*_max_	48.35 ± 16.35	49.48 ± 16.37	47.75 ± 17.03	0.041	0.624	0.274
	*D*_mean_	42.24 ± 16.05	43.25 ± 16.55	41.02 ± 16.85	0.138	0.294	0.016

OARs: organs at risk; PRV: planning risk volume; Len-L: left lens; Len-R: right lens; Optic nerve-L: left optic nerve; Optic nerve-R: right optic nerve; IMRT: intensity-modulated radiation therapy; VMAT: volumetric-modulated arc therapy; HT: helical tomotherapy.

## Data Availability

The data used to support the findings of this study have been deposited in the DRYAD repository (10.5061/dryad.b2rbnzs94)
